# The Role of DNA Methylation in Common Skeletal Disorders

**DOI:** 10.3390/biology1030698

**Published:** 2012-11-22

**Authors:** Jesús Delgado-Calle, José A. Riancho

**Affiliations:** Department of Internal Medicine, H.U. Marqués de Valdecilla-IFIMAV-University of Cantabria, Santander 39008, Spain; Email: jesusdelgadocalle@gmail.com

**Keywords:** gene expression, epigenetics, genetics, osteoporosis, osteoarthritis

## Abstract

Bone is a complex connective tissue characterized by a calcified extracellular matrix. This mineralized matrix is constantly being formed and resorbed throughout life, allowing the bone to adapt to daily mechanical loads and maintain skeletal properties and composition. The imbalance between bone formation and bone resorption leads to changes in bone mass. This is the case of osteoporosis and osteoarthritis, two common skeletal disorders. While osteoporosis is characterized by a decreased bone mass and, consequently, higher susceptibly to fractures, bone mass tends to be higher in patients with osteoarthritis, especially in the subchondral bone region. It is known that these diseases are influenced by heritable factors. However, the DNA polymorphisms identified so far in GWAS explain less than 10% of the genetic risk, suggesting that other factors, and specifically epigenetic mechanisms, are involved in the pathogenesis of these disorders. This review summarizes current knowledge about the influence of epigenetic marks on bone homeostasis, paying special attention to the role of DNA methylation in the onset and progression of osteoporosis and osteoarthritis.

## 1. Bone Cells and Bone Remodeling in Health and Disease

**Figure 1:**
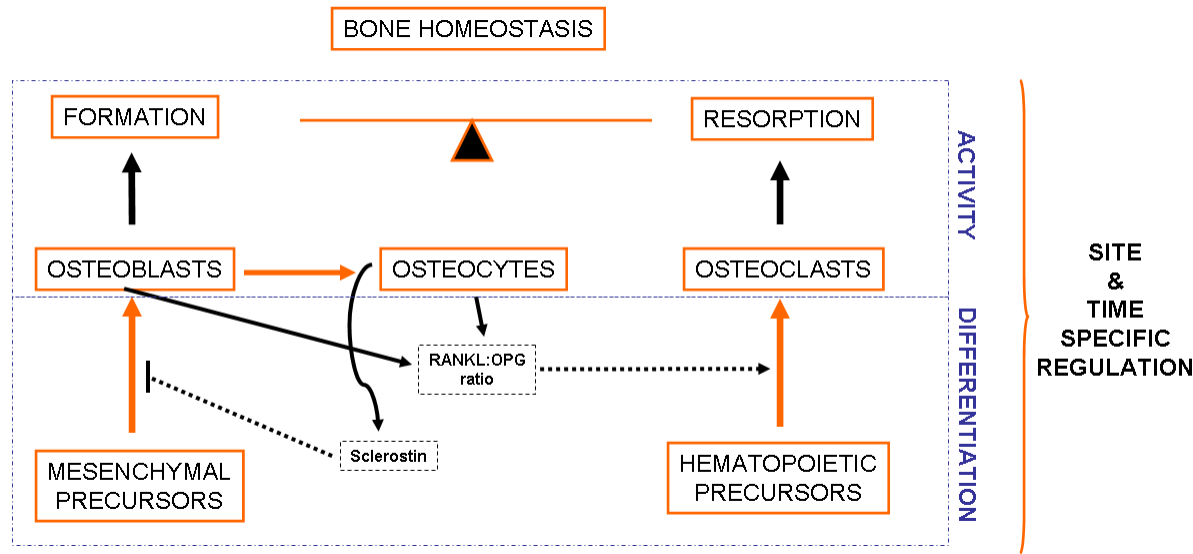
Bone is a complex and dynamic organ, which is constantly being remodeled by the balanced and coupled activity of the cells present in this tissue. Osteoblasts derive from mesenchymal precursors and eventually evolve into osteocytes and lining cells. Cells of the osteoblastic lineage are responsible for bone formation. Osteoclasts, derived from hematopoietic cells, are responsible for bone resorption. Interestingly, osteoclast differentiation is influenced by soluble factors secreted by osteoblasts and osteocytes, especially those related to the RANKL-RANK signaling pathway. On the other hand, osteocyte-derived sclerostin negatively modulates bone formation.

Bone is a complex connective tissue composed of a calcified extracellular matrix in which different cell types are embedded. The complexity of bone is particularly evident in the cells present in this tissue and the multifaceted interactions between them [1]. Bone cells belong to two different families: the osteoblastic and the osteoclastic families (see Figure 1) [2,3].

There are several cell types within the osteoblastic lineage, including osteoblasts, osteocytes and lining cells. All of them derive from mesenchymal precursors that differentiate into osteoblasts, which eventually evolve into osteocytes or lining cells [4]. Cells of the osteoblastic lineage modulate the proliferation and differentiation of cells belonging to the osteoclastic lineage, mainly by the RANKL-OPG-RANK signaling pathway [5,6,7]. Osteocytes derive from some osteoblasts that become embedded and surrounded by bone matrix [8]. These cells are emerging as the responders to mechanical stimuli that regulate bone formation and resorption, as well as key regulators of bone metabolism. Osteocytes modulate bone turnover through the modulation of the Wnt pathway and other pathways that influence the activity of both osteoblasts and osteoclasts [9,10,11]. 

Osteoclasts derive from hematopoietic precursors. These cells are formed by the fusion of cells of the monocyte-macrophage lineage and are responsible for bone resorption. As mentioned before, osteoclastogenesis is influenced by osteoblasts and osteocytes, which produce several factors critical for the differentiation of osteoclast precursors, such as the receptor activator of nuclear factor kappa-B ligand (RANKL) and the macrophage colony-stimulating factor (MCS-F) [7]. RANKL interacts with RANK (Receptor activator of nuclear factor kappa-B), present in the membrane of osteoclastic precursors, promoting the activation of the Nuclear factor Kappa B, which induces cell fusion and differentiation to originate multinucleated mature osteoclasts [12,13]. It is important to note that osteoblastic cells also express osteoprotegerin (OPG), which acts as a soluble decoy receptor for RANKL, thus impairing the RANKL-RANK interaction [14]. Mature osteoclasts locate on specific surfaces of bone, where they break down and resorb bone matrix, replacing old bone with new bone [12].

Bone is under constant turnover throughout life in order to maintain its properties. It is believed that this process, known as bone remodeling, occurs, in part, randomly. However, sometimes, osteocytes can mark the site where a remodeling cycle must be started in order to repair microcracks, which represent the so called “targeted remodeling” [15,16]. Bone remodeling is carried out by the cyclic and coupled activity of osteoclasts and osteoblasts. The process starts when osteoclast precursors are recruited to the bone surface, where they differentiate and remove a small volume of bone. Then osteoclasts undergo apoptosis and osteoblasts arrive to the region to fill up the defect with new bone [17]. Bone mass homeostasis depends on the balance between bone formation and bone resorption. Any disequilibrium between bone formation and bone resorption leads to changes in bone mass. This is the case of osteoporosis and osteoarthritis, two common skeletal diseases that tend to show changes of bone mass in opposite directions [18].

The maintenance of bone mass requires proper cell differentiation and cell activity. Osteoblast and osteoclast differentiation processes are highly organized and driven by deep changes in the gene expression patterns that in turn result in cells with different shapes and functions [3,19]. Since bone remodeling requires the sequential action of osteoclasts and osteoblasts at a given region, the differentiation of both cell types is controlled in a time and site-specific manner, and influenced not only by intrinsic factors, but also by some systemic and environmental factors. Emerging lines of evidence suggest that epigenetic mechanisms play an important role in establishing cellular identities and controlling gene expression. Herein, we summarize the current knowledge about the role of epigenetics, and specifically DNA methylation marks, in bone homeostasis and pathogenesis.

## 2. DNA Methylation Influences Gene Expression

Presently, epigenetics is defined as, “The study of stable genetic modifications that results in changes in gene expression without a corresponding alteration in DNA sequence [20]”. Importantly, epigenetic marks integrate intrinsic and environmental stimuli and confer both lineage commitment and phenotypic plasticity [21]. Thus, epigenetic marks could be considered as a link between genotype, environment, phenotype and disease. Epigenetic mechanisms comprise DNA methylation, post-translational histone modifications and non-coding RNAs [22,23]. Although the mechanisms of epigenetic inheritance during cell division are well established, heritable epigenetic patterns from parents to offspring are starting to be revealed [24,25].

In eukaryotes DNA methylation consists in the covalent addition of methyl groups to cytosines that precede guanines (CpG) [26]. In vertebrate genomes, CpG sites are predominantly methylated [27]. However, the globally methylated pattern is disrupted in regions known as CpG islands. These areas are present in approximately 70% of gene promoters. On average, CpG islands are 1 kb long, have an elevated C + G content and are frequently demethylated [28]. DNA methylation variations do not occur exclusively at CpG islands. Recently, it has been also shown that methylation occurs at a short distance from the CpG islands (at “CpG island shores”), rather than in the islands themselves [29]. The term CpG island shore refers to regions of lower CpG density that lie in the vicinity (~2 kb) of CpG islands. The addition of methyl groups to cytosines is catalyzed by DNA methyltransferases (DNMTs) [30]. DNMT1 was the first methyltransferase identified in mammals [31]. DNMT1 maintains DNA methylation during cell division by reading and copying the pattern in the hemimethylated strand [31]. Other members of the family are DNMT3A, DNMT3B, responsible for *de novo* methylation [32], and DNMT2, which has been recently shown to methylate tRNAs [33]. DNA methylation is considered an efficient repressor of transcriptional activity. Until recently, it was commonly thought that methyl groups directly prevent the binding of essential transcription factors to their targets. Although this is true for a specific set of transcription factors, it is not a general phenomenon. In fact, the binding sites for many transcription factors do not have CpGs. Emerging evidences support that the presence of methyl groups models the surrounding chromatin, inducing a DNA conformation less accessible to the transcription machinery. The mechanisms underlying this packing change are not fully understood yet, but many studies have concentrated on nucleosome structure, methyl-binding proteins, such as MECP2, MBD2 and MBD3, and interactions with chromatin remodeling enzymes [34,35,36]. Whatever the mechanism, on the basis of its potential to silence promoters, DNA methylation is supposed to play an important role in cell commitment and cell-specific gene expression.

**Figure 2:**
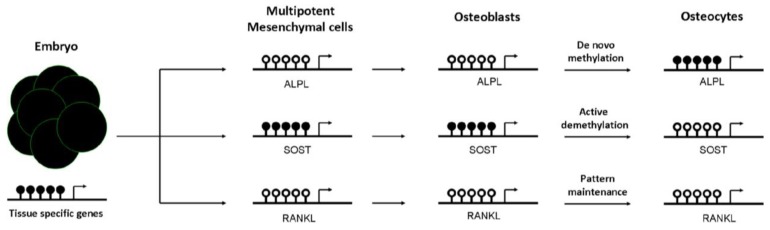
Dynamics of tissue-specific DNA methylation of bone cells. DNA methylation is established early in the embryo. Generally, at this stage, pluripotent genes and house-keeping genes are unmethylated, whereas tissue-specific genes are largely methylated. Then, the differentiation to mesenchymal cells promotes not only demethylation of many of these specific cell lineage genes, but also *de novo* methylation events to silence multipotent-specific genes. During mesenchymal commitment to the osteoblastic lineage, some unmethylated genes undergo *de novo* methylation to be silenced in osteocytes, such as alkaline phosphatase gene (*ALPL*). However, other methylated genes are actively demethylated to promote its expression in the late osteocyte [sclerostin (*SOST*)]. Lastly, other genes already demethylated in mesenchymal cells remain demethylated in differentiated cells, such as the Receptor activator of nuclear factor kappa-B ligand (*RANKL*). Black and white circles represent DNA methylation and hypomethylation, respectively.

How methylation is specifically targeted to a subset of promoters is generating intense debate. Apparently, methylation patterns are established early in the embryo [32,37,38]. Several studies support the idea that the ultimate methylation profile is determined by the underlying DNA sequence. In this sense, it has been shown that local DNA sequence is one of the main determinants for targeting DNA methylation to a specific locus. Thus, sequence variation between individuals might contribute to differential methylation patterns [38,39]. In fact, recent findings suggest that allele-specific methylation (ASM) is a common feature across the genome [40,41]. Notably, most of the ASM is strongly associated with SNPs genotypes [42,43]. Subsequent changes in the methylation pattern, generally of a tissue-specific nature, occur following implantation (*i.e*., repression of pluripotent genes) [44,45]. It has been proposed that tissue-specific changes occur through mechanisms apparently recruiting molecules needed for *de novo* methylation and demethylation (see Figure 2). Whereas demethylation may occur by active or repairing mechanisms [46,47], *de novo* methylation may be mediated by polycomb complexes [48].

## 3. Role of DNA Methylation in Establishing a Bone Cell Phenotype

An impaired mesenchymal differentiation negatively affects bone mass. Several studies suggest that the osteogenic capacity of mesenchymal cells decrease with aging [49]. However, the contribution of this event to the decline of bone mass associated with aging is not clear yet. Osteogenic differentiation of mesenchymal cells towards osteoprogenitor and osteoblastic cells is regulated by several mechanisms, including DNA methylation. Kang *et al*. demonstrated that promoter methylation changes during mesenchymal cell differentiation [50]. Likewise, it has been proposed that active demethylation of gene promoters (*i.e.*, osteocalcin, osterix, or *Runx*2), via GADD45-dependent mechanisms, is involved in the ostegenic differentiation of mesenchymal cells [51]. In fact, as reported by Locklin *et al*., osteogenic differentiation may be modulated by demethylating agents [52]. DNA methylation marks are not only important in osteoblastogenesis, but also afterwards in the osteoblast to osteocyte transition. Our group demonstrated that CpG methylation at regulatory regions controls the expression of the alkaline phosphatase and sclerostin genes. We observed that the hypermethylation of *ALPL* and *SOST* promoters was inversely correlated with gene expression in osteoblastic cells. Furthermore, we showed that the presence of methyl groups at the proximal promoter of *SOST* markedly decreased the transcriptional activity of this sequence, presumably by impairing the binding of essential transcription factors to the core promoter. In addition, we demonstrated that the methylation of those promoters changes during osteoblast differentiation towards osteocytes and controls gene expression in a cell-specific manner [53,54]. DNA methylation at *ALPL* promoter increased progressively during osteoblast differentiation, silencing *ALPL* expression in osteocytes. DNA methylation represses *SOST* expression in osteoblasts, whereas the physiological demethylation of its promoter favored the expression of this gene in osteocytes (see Figure 2). Consistent with this observation, *SOST* promoter remains methylated in other cell types that do not express sclerostin [53]. The results of other investigators also support that the expression of a number of genes important for osteogenic differentiation and osteoblast/osteocyte activity (including podoplanin, osteopontin, Brachury transcription factor, estrogen receptor, aromatase, collagen cross-linking enzyme lysyl oxidase or the homeobox protein *Dlx-5*) is regulated by DNA methylation [55,56,57,58,59,60,61,62].

On the other hand, DNA methylation contributes to regulate self-renewal and differentiation capacity of hematopoietic cells, the early precursors of osteoclasts [63]. The subsequent differentiation of osteoclast precursors towards mature osteoclasts is tightly regulated by soluble factors secreted by osteoblasts and osteocytes, such as RANKL, OPG and MCS-F [7,64]. As first reported by Kitazawa *et al*., and recently confirmed by our group in human samples, *RANKL* and *OPG* expression is regulated by DNA methylation in osteoblastic cells [65,66]. CpG methylation at the regulatory regions of *RANKL* and *OPG* genes is associated with low transcript levels. In turn, the demethylation of their promoters mediated by 5-azadeoxycitidine, a demethylating agent, induces the expression of both genes. Based upon these evidences, epigenetic mechanisms appear to be important for osteoclast differentiation (reviewed by Yasui *et al*. [67]). However, it is worth emphasizing that besides DNA methylation, other epigenetic marks, such as microRNAs or chromatin modifiers, are also involved in determining the differentiation and activity of bone cells, recently reviewed by Delgado-Calle *et al*., Kato S *et al*. and Earl CS *et al*. [68,69,70].

## 4. Methylation Marks and Common Skeletal Diseases

### 4.1. Osteoporosis

Osteoporosis is characterized by reduced bone mass and/or abnormal bone microarchitecture, which decrease bone strength and augment the susceptibility to fracture. Common osteoporotic fractures include those of the vertebral bodies, hip, pelvis, proximal arm and wrist. Any imbalance in the activity of osteoclasts and osteoblasts in a way such that bone formation is smaller than bone resorption results in osteoporosis. In some cases, osteoporosis is secondary to an underlying disease, but quite often it is just an exaggeration of the universal age-associated decrease in bone mass. In part, it is related to the diminished availability of sex steroids taking place in women after menopause and in elderly men [71], but other incompletely known factors associated with aging are likely involved [49,72]. Whatever those mechanisms might be, osteoporosis is the final result of the complex interplay between genetic and acquired factors in which epigenetic marks may also participate.

As discussed above, experimental evidence shows that the methylation of several genes play a major role in the differentiation of bone cells, which is required to sustain a normal bone remodeling. Therefore, it might be hypothesized that DNA methylation is involved in the pathogenesis of osteoporosis, though there is little evidence directly supporting this hypothesis so far. However, it has been recently proposed that reduced *Dnmt1* activity decreases bone mineral density and body weight [73]. A number of studies suggest that environmental influences may contribute to shaping the methylation pattern of the individual and that the pattern may change in association with aging [74,75,76,77,78]. Several animal studies have related the environmental factors during early phases of development with DNA methylation and skeletal status. In fact, maternal dietary intake has been shown to influence bone mass of the offspring, both in experimental animals and in humans [79]. In some cases, DNA methylation may be involved. For instance, dietary restriction of pregnant rats induces changes in the methylation of genes that are important for bone cell differentiation and activity, such as the glucocorticoid receptor and the peroxisomal-activated receptor genes [80,81,82,83].

### 4.2. Osteoarthritis

Whereas osteoporosis is primarily a bone disorder, osteoarthritis is the most common form of joint disease. In fact, damage of the articular cartilage is the hallmark of osteoarthritis. However, osteoarthritis involves an abnormal remodeling of other tissues in affected joints, such as the synovium and the subchondral bone [84,85]. Thus, other typical changes in the joints of patients with osteoarthritis (besides the narrowing of joint space secondary to cartilage thinning), include the formation of osteophytes (bone excrescences at the periphery of the joints) and sclerosis of the subchondral bone. The extracellular matrix of the articular cartilage contains several types of collagen (II, IX and XI) and proteoglycanes, such as aggrecan. Several investigators have shown an altered homeostasis in the diseased cartilage, with increased expression of catabolic genes, accompanied by a diminished synthesis of components of the cartilaginous matrix. Matrix metalloproteases (MMPs) and aggrecanases (ADAMTS-4 and 5) are regarded as major enzymes mediating the destructive process of cartilage. The sclerosis of subchondral bone used to be considered a secondary reactive change, but in recent years, the concept is emerging that subchondral bone may play more than a passive role in the pathogenesis of the disease. In line with this concept, it has been postulated that cytokines released by bone cells influence the activity of chondrocytes, and vice-versa [86]. Furthermore, subchondral bone influences the overlying cartilage, not only through its biomechanical properties, but also through the synthesis of various humoral factors (see recent reviews [86,87,88,89,90]).

After the seminal work by Roach *et al*. [91,92], it has been demonstrated that, in some cases, changes in chondrocyte gene expression are associated with inverse changes in DNA methylation. For instance, Zimmerman *et al*. reported that the induction of type X collagen expression during chondrogenic differentiation of mesenchymal stem cells is associated with the demethylation of specific CpGs in its promoter [93]. However, in adult chondrocytes, the promoter was methylated, which correlated with the absence of gene expression. Inverse correlations between methylation and gene expression have been reported for other genes, including osteogenic protein 1 (*OP-1*), interleukin 1 beta, *MMP3*, *MMP9*, *MMP13*, leptin and *ADMTS4* [58,94,95]. The exact molecular mechanisms have not been completely elucidated, but in some cases they may include methylation-dependent differences in the ability to recruit transcription factors, such as CREB, to gene regulatory regions [96]. However, gene expression and DNA methylation are not always inversely correlated. Thus, the reduced expression of genes, such as type II collagen or aggrecan reported in diseased cartilage, does not seem to be associated with increased methylation of these genes [94,97]. On the other hand, the functional consequences of gene methylation have also been revealed by *in vitro* experiments, showing that inducing DNA demethylation by 5-azadeoxycitidine modulates the differentiation of articular chondrocytes [98]. 

Some data suggest that the shape of the bones may also influence osteoarthritis. For instance, epidemiological studies showed that the certain morphological characteristics of the femoral epiphysis and the pelvis determine the risk of hip osteoarthritis [99,100,101]. This has given support to the hypothesis of a developmental origin of osteoarthritis [102,103,104]. The importance of developmental factors is also emphasized by recent results from genome-wide association studies [105,106]. Somewhat unexpectedly, these studies have not revealed significant associations between osteoarthritis and genes typically involved in cartilage homeostasis, such as those encoding proteases. Instead, they have shown association between some polymorphisms of genes involved in joint development, such as *GDF5*, and osteoarthritis of the large joints, particularly the knee and the hip [107,108]. It is currently thought that the epigenome is the consequence of environmental factors, genetic features and stochastic variations. In this regard, it is interesting to note that some *GDF5* single nucleotide polymorphisms (SNPs) showed differential allelic expression. Interestingly, the functional effect of these “CpGs SNPs” on gene expression is modulated by DNA methylation [108].

Our group has recently published a genome-wide methylation study in bone samples obtained from patients with severe hip osteoarthritis and compared the results with those obtained in patients with osteoporotic hip fractures [109]. Our results revealed several genes showing differential methylation between osteoporotic and osteoarthritic patients. Somewhat unexpectedly, genes showing differential methylation where not typical bone candidates. However, functional network analysis revealed that epigenetic changes in “upstream” regulatory genes may have downstream influences on well-known bone-related genes. Interestingly, and in tune with the developmental hypothesis, we found that genes showing differential methylation were overrepresented in pathways related to skeletal development, and particularly those of the homeobox family. Globally, DNA methylation correlated negatively with gene expression in both groups of patients. However, as observed in other studies, we found a subset of genes in which there was no inverse correlation between DNA methylation and the abundance of gene transcripts, thus pointing out that factors other than methylation play an important role in the fine tuning of gene expression in adult tissues.

### 4.3. Tumors and Bone

Some tumors originate in the skeleton, and many others have a propensity to metastasize in bone. In fact, bone metastases are very common in a variety of advanced cancers and contribute importantly to cancer morbidity. The mechanisms influencing the metastatic potential of tumor cells and their tropism for certain tissues are being actively investigated. They are likely complex and include factors related to the tumor itself and others which depend on the tissue hosting the metastases. In some cases, they may include certain methylation patterns that result in specific gene expression signatures that facilitate the initiation or the growth of the metastases. Prostate cancer cells are among those with a stronger tropism for bone. Saha *et al*. reported that the hypomethylation of the E-cadherin gene, and the subsequent reduction of gene expression, was associated with metastatic prostate cancer cells in bone [110]. Also, reduced DNA methylation is associated with increased expression of the parathyroid hormone-related protein (PTHrP) by breast cancer cells, which in turn may facilitate osteolysis and the growth of the metastatic niche [111]. On the other hand, the fact that certain tumors have specific gene methylation patterns, different from those of normal tissues, has raised the possibility of using the analysis of DNA remnants present in serum as a biomarker to help in diagnosing specific types of cancer. 

## 5. Concluding Remarks

**Figure 3:**
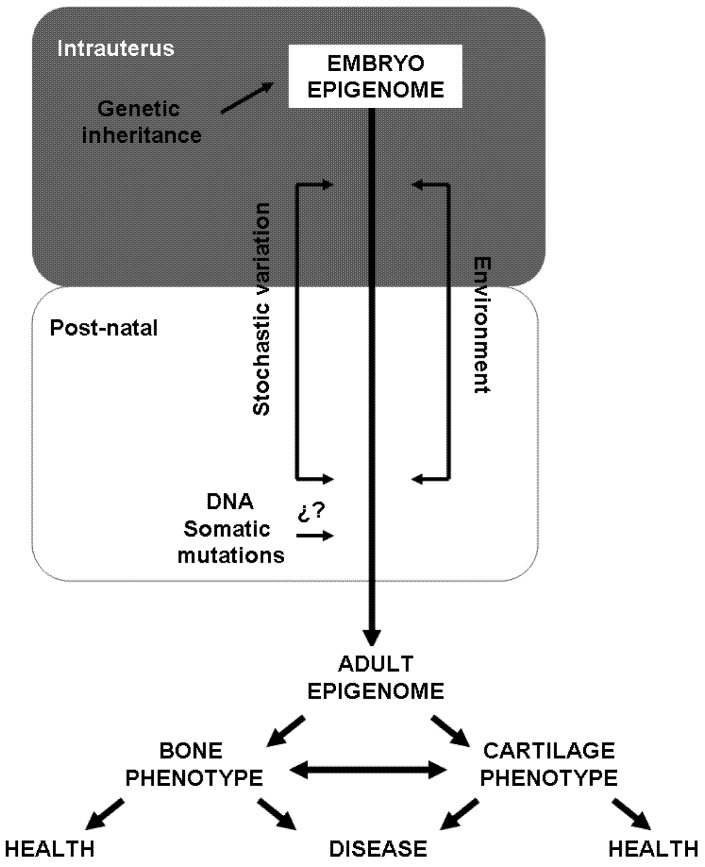
Factors involved in epigenetic variability. Epigenetic marks can change throughout life and, thereby, determine the adult phenotypes of the cells present in bone. Several factors (*i.e.*, starving) may influence epigenetic marks at different stages of intrauterus life. For instance, genetic inheritance might influence the *de novo* methylation that occurs before implantation. Likewise, stochastic variations and environmental cues may also influence the epigenetic pattern. During the post-natal life, epigenetic variability may depend on environmental (*i.e.*, toxic habits, food...) and intrinsic factors (genetic predisposition), as well as on the occurrence of DNA somatic mutations. Epigenetic signatures are directly linked to the control of cell differentiation and gene expression, thus accumulated epigenetic variability can lead to aberrant phenotypes and, consequently, induce skeletal disease.

Common skeletal disorders, such as osteoporosis and osteoarthritis, are the result of a complex interplay of genetic and acquired factors. However, despite tremendous efforts, including several GWAS, the genetic factors so far identified explain less than 10% of the genetic risk. This suggests that mechanisms not related to DNA sequence may be involved in the development of these diseases. This could be the case of DNA methylation and its mediators. DNA methylation marks are heritable, at least through cell divisions, control gene response to environment, change with aging and underlie cell commitment and the spatiotemporal control of gene expression. DNA methylation plays an important role in the differentiation of cells of the osteoblastic and osteoclastic lineages. Therefore, it is tempting to speculate that the aberrant phenotypes observed in bone diseases might be the consequence of a combination of intrinsic and environmental factors, including gene sequence variations and epigenetic signatures (see Figure 3). However, it is important to note that DNA methylation is only one of the mechanisms underlying gene expression. Thus, the integration of knowledge from both epigenenomics and genomics, together with other “omics” (*i.e.*, transcriptomics, proteomics) will be essential for the full understanding of the underlying mechanisms that govern the initiation and progression of bone diseases. Although still a long way to go, further studies in bone epigenetics may open a new door for drug development combining genetic and epigenetic strategies.
